# PromGER: Promoter Prediction Based on Graph Embedding and Ensemble Learning for Eukaryotic Sequence

**DOI:** 10.3390/genes14071441

**Published:** 2023-07-13

**Authors:** Yan Wang, Shiwen Tai, Shuangquan Zhang, Nan Sheng, Xuping Xie

**Affiliations:** 1 Key Laboratory of Symbolic Computation and Knowledge Engineering of Ministry of Education, College of Computer Science and Technology, Jilin University, Changchun 130012, China; wy6868@jlu.edu.cn (Y.W.); taisw21@mails.jlu.edu.cn (S.T.); shengnan21@mails.jlu.edu.cn (N.S.); xiexp21@mails.jlu.edu.cn (X.X.); 2 School of Artificial Intelligence, Jilin University, Changchun 130012, China; 3 School of Cyber Science and Engineering, Nanjing University of Science and Technology, Nanjing 210094, China

**Keywords:** promoter prediction, sequence analysis, graph embedding, ensemble learning, interpretability

## Abstract

Promoters are DNA non-coding regions around the transcription start site and are responsible for regulating the gene transcription process. Due to their key role in gene function and transcriptional activity, the prediction of promoter sequences and their core elements accurately is a crucial research area in bioinformatics. At present, models based on machine learning and deep learning have been developed for promoter prediction. However, these models cannot mine the deeper biological information of promoter sequences and consider the complex relationship among promoter sequences. In this work, we propose a novel prediction model called PromGER to predict eukaryotic promoter sequences. For a promoter sequence, firstly, PromGER utilizes four types of feature-encoding methods to extract local information within promoter sequences. Secondly, according to the potential relationships among promoter sequences, the whole promoter sequences are constructed as a graph. Furthermore, three different scales of graph-embedding methods are applied for obtaining the global feature information more comprehensively in the graph. Finally, combining local features with global features of sequences, PromGER analyzes and predicts promoter sequences through a tree-based ensemble-learning framework. Compared with seven existing methods, PromGER improved the average specificity of 13%, accuracy of 10%, Matthew’s correlation coefficient of 16%, precision of 4%, F1 score of 6%, and AUC of 9%. Specifically, this study interpreted the PromGER by the t-distributed stochastic neighbor embedding (t-SNE) method and SHAPley Additive exPlanations (SHAP) value analysis, which demonstrates the interpretability of the model.

## 1. Introduction

A promoter is a short, non-coding sequence region on a genomic DNA that serves as the starting signal for gene transcription [1]. Typically located at the 5’ end of the gene, the promoter can be bound by RNA polymerase II to initiate transcription under the regulation of transcription factors [2]. The biological function of the promoter is to activate gene transcription, leading to the expression of the corresponding protein or RNA. In other words, the promoter can allow genes to be transcribed at specific times, locations, and levels, thus affecting protein synthesis. In eukaryotes, promoters generally consist of multiple nucleotide sequences, including the core promoter, enhancers, and silencers, which regulate the transcriptional activity of the gene by binding to transcription factors. The core promoter is the most basic part of the promoter, consisting of the transcription start site (TSS) and conserved sequence elements, such as the TATA box, CAAT box, and GC box [3]. The TATA box is the most typical and ancient core promoter element, which is present in organisms ranging from yeast to plants and metazoans. Located about 25–35 base pairs upstream of the TSS with the consensus sequence like ‘TATAAA’, the TATA box defines the direction of transcription and indicates the DNA strand to be read [4]. Moreover, the GC box and CAAT box are the upstream activating elements located at −40 to −110 bps, which regulates the rate of transcription initiation [5,6]. These elements can attract different transcription factors and work together to regulate gene transcription, closely linked to human diseases, such as tumors [7], coronary artery disease [8], and diabetic nephropathy [9]. Therefore, the prediction of promoters plays a vital role for the annotation of the complete structure of a gene, thereby influencing cell growth, differentiation, and adaptation for further understanding the mechanisms of gene transcription and expression regulation [10].

Traditional biological experiment approaches to predict promoters are mutational analysis [11] and immunoprecipitation assays [12,13]. With the progression in high-throughput whole-genome sequencing [14], it dramatically reduced sequencing costs, and gene sequence data for more species are available. Therefore, the computational feature extraction methods are more satisfying in terms of efficiency, labor, and expenses. The main difference among existing computational methods is the way to extract sequence features [15]. Approaches to extracting local features can be categorized into three types: the GpG-based method, the content-based method, and the signal-based method. The GpG-based method relied on the location of GpG islands as a vital feature in identifying promoter regions, given that the first exon region in human genes usually contains GpG islands [16]. However, only around 60% of promoters actually contain GpG islands, resulting in unfavorable outcomes. The content-based method utilized a k-length window to construct k-mer basic unit frequency, including the position-specific approach [17], variable length substrings approach [18], word frequency approach [19], and other counting approaches. However, these methods ignore the potential impact of core promoter elements and the spatial information provided by the base pairs in sequences. The signal-based method primarily focused on element information in sequences, namely, finding promoter elements associated with RNA polymerase binding sites. The most commonly used elements are the initiator (Inr) [20], CCAAT-box [10], GC-box [10,21], and TATA-box [10,20,22]. Since it ignores the non-element aspects of the sequence, this method is generally not employed alone.

At present, more and more computational methods tend to study the whole promoter sequence information, not limited to local elements. The consensus of these methods is to explore the entire sequence data, extract relevant and valuable information, make accurate decisions, and promote knowledge discovery [23,24]. For example, in the traditional machine-learning field, this category requires domain-knowledge-related features to obtain information from DNA sequences, involving logistic regression (LR) [25], a support vector machine (SVM) [26,27], a hidden Markov model (HMM) [28,29], linear discriminant analysis (LDA) [30], a decision tree (DT) [31], a random forest (RF) [32], an artificial neural network (ANN) [33], etc. In contrast to machine learning, deep-learning methods mines potential information without any domain knowledge in constructing the feature vector. Through multiple layers of feature representations from the raw biological sequence data (i.e., DNA, RNA, or protein sequences), it has been increasingly implemented as a mainstream model in the promoter prediction field [34,35,36]. For instance, CNNProm [37] employed a convolutional neural network (CNN) to predict eukaryotic promoter sequences on TATA-containing promoter data and TATA-less promoter data. To further capture long-term dependencies of sequential samples, DeePromoter [38] combined a bidirectional long short-term memory (BiLSTM) with CNN, which significantly reduced the number of false-positive predictions. In addition, Depicter [39] proposed a CNN coupled with capsule layers to train and optimize the prediction model. By the application of dynamic routing instead of the maximum pooling or averaging pooling, Depicter balanced core elements with position information. In summary, most deep-learning methods for promoter prediction employed the CNN algorithms, demonstrating the ability to capture promoter sequence characteristics.

Despite mainstream deep-learning methods having achieved effective advances, several issues still need to be addressed. Firstly, deep-learning methods usually take one-hot encoding as the model input, mostly. Due to sparse and high dimensionality, it is unable to sufficiently extract the biological semantic information of the nucleotide. Secondly, there are repeated structures in eukaryotic promoter sequences called promoter elements. Although promoter elements are widely observed in eukaryotes, it is not the only basis for predicting promoter sequences. By the convolutional operation, deep-learning models will rely on these shallow features, actually, ignoring the deeper non-element information of a promoter sequence [38]. Meanwhile, pooling is another major procedure in CNN methods. However, the distribution of nucleotide location can be affected by this compression [39]. Thirdly, since there are more non-promoter samples than promoter samples, the class between the positive and negative is imbalanced. Moreover, the promoter sequence samples are significantly smaller compared with the data size in other domains. Thus, when simply utilizing deep-learning methods, the promoter samples’ class imbalance and small sample sizes cause the model to be easily overfit [40]. Finally, as a feed-forward network, the input is a fixed length, which limits the generalization ability of the model. The input promoter sequences are also assumed to be mutually independent, ignoring the fact that there are potential relationships between promoter sequences.

In the past few years, graph deep learning was raised to represent the biological network data in various deep-learning methods. It had gained popularity in the computational biology area, such as graph generation, link prediction, and node classification [41]. Compared with other models in deep learning, the natural advantage of graph deep learning in capturing hidden information brings new opportunities to design computational models in the biology field [42]. In promoter prediction, the global information relationships among promoter sequences can be described by the graph data structure. Constructing the graph, graph deep learning may be able to capture deeper feature representations. Meanwhile, ensemble learning is a traditional machine-learning technique that effectively solves the challenges of small sample size, high dimensionality, and data noise, which is also employed in models for promoter prediction [43]. With its architectures and strategies, ensemble learning leads to remarkable and widespread breakthroughs in the field of bioinformatics [44,45,46]. 

In this study, we developed a promoter sequence prediction model called PromGER to predict eukaryotic promoters for multi-species via combining graph-embedding methods and ensemble learning. The main contributions of the paper are summarized as follows: (1) We extract a typical sequence’s local features to illustrate biological attributes of nucleotides, including the nucleotide chemical property (NCP), nucleotide density (ND), electron–ion interaction pseudopotential (EIIP), and bi-profile Bayes (BPB) features.(2) The promoter sequences are modeled as a graph, where nodes are the promoter sample sequences and edges represent potential contacts among sequences, aiming at the ability to obtain global information. (3) Three graph-embedding methods from various scales, involved with the single node, group community, and global structure, are applied to extract the relationship representation features within the graph. The features are further integrated into a popular tree-based ensemble-learning framework named CatBoost to train the classifier. (4) We evaluated and verified the prediction performance of our model by the ablation study, t-SNE method, and SHAP value. Compared with different promoter prediction models on independent test datasets, the results indicated the effectiveness of PromGER for promoter prediction.

## 2. Materials and Methods

### 2.1. Overall Framework

**Figure 1:**
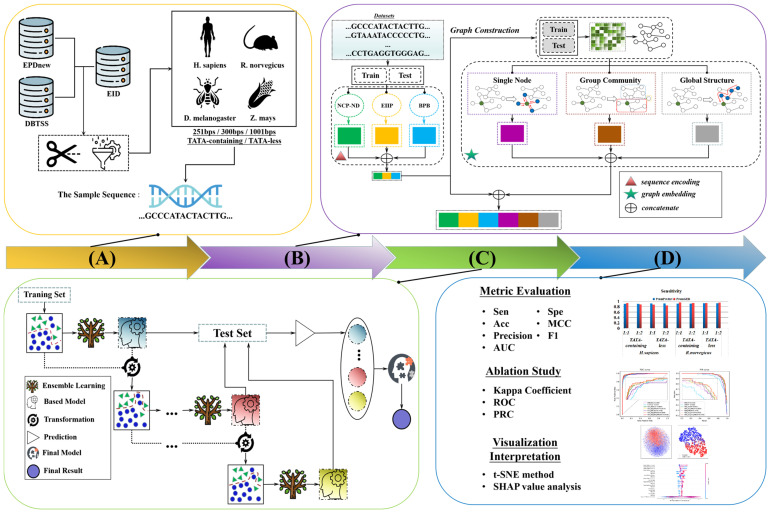
The developmental flowchart of PromGER: (**A**) data collection; (**B**) feature extraction; (**C**) ensemble learning; and (**D**) performance evaluation and interpretability analysis. In the graph embedding, the red line or square represents various scales, involved with the single node, group community, and global structure. In the ensemble learning, the green triangle represents the positive sample, and the blue triangle represents the negative sample.

The overall framework of PromGER mainly includes four steps. In the first step, we collect reliable datasets. In the second step, we first performed a train–test split due to the features of BPB. Then, we introduced multiple sequence features to encode the promoter accordingly, including NCP, ND, EIIP, and BPB. In this feature calculation phase, it is noted that we used the entire data for one species, including both train and test. In the graph construction stage, we still used the entire data for constructing the graph, including train and test, so that the subsequent graph embedding can take place. In the third step, PromGER employ an ensemble-learning framework to train the prediction model based on the previously split data. The last step evaluates the prediction performance of PromGER on independent test datasets, designs the ablation study, and provides an interpretability analysis of PromGER via the t-SNE and SHAP viewpoint. The framework of the PromGER is shown in Figure 1.

### 2.2. Dataset Collecting

Constructing a proper and standard dataset is a fundamental step in designing a robust prediction model [47]. In this study, we used the eukaryotic data organized by Zhang et al. [48], which contain comprehensive and up-to-date datasets for multiple species and support assessment research on eukaryotic promoters. For the PromGER, the promoter prediction is a binary classification task, where the promoter samples can be divided into positive samples and negative samples. The positive samples are promoter sequences labeled as 1, while the negative samples are non-promoter sequences labeled as 0. The positive sample resources are collected from the Eukaryotic Promoter Database (EPDnew) and the DataBase of Transcriptional Start Sites (DBTSS). EPDnew [49] is an annotated nonredundant collection of eukaryotic POL II promoters, for which the transcription start site has been discovered experimentally. DBTSS [50] is also a database determining the biological information of TSS. Meanwhile, negative sample datasets are obtained from Exon-Intron Database (EID) [51], which documents the exon and intron information of the corresponding species. 

To assess the relative functionality of alternative approaches objectively, this study chose promoter sequences from *Homo sapiens* (*H. sapiens*) and *Rattus norvegicus* (*R. norvegicus*), which are widely applied to existing promoter predictors. In eukaryotic promoter prediction, the promoter sequences are usually categorized into TATA-containing types (i.e., promoter sequences with the TATA box element) and TATA-less types (i.e., promoter sequences without the TATA box element) by most models. Usually, the lengths of input sequences (251, 300, or 1001 bps) are not uniform among existing promoter predictors. Therefore, the input of the predictors was spliced to the corresponding sequence length by extracting from upstream to downstream regions of TSS. For each species, the CD-HIT-EST tool [52] with an identity threshold of 0.8 [53] was employed to exclude redundancy sequences. 

In the above manner, there are two TATA types for each species, including TATA-containing and TATA-less. Moreover, the input sample length of every TATA type can be divided into 251 bps, 300 bps. and 1001 bps. For the balanced dataset, the non-promoter sequences had the same amount as the promoter sequences. The final statistical results of the balanced datasets in this paper are shown in Table 1. Generally, the eukaryotes have more non-promoters than promoters, which means the evaluation on the imbalanced datasets is essential for PromGER. To compare model performance fully, we have also selected corresponding imbalanced datasets for *H. sapiens* and *R. norvegicus*, which include more negative samples than positive samples. The specific numbers in the imbalanced datasets are shown in Table 2. 

Moreover, we used *Drosophila melanogaster* (*D. melanogaster*) and *Zea mays* (*Z. mays*) datasets with 300 bps for ablation study and interpretability analysis, respectively, as shown in Table 1 and Table 2. The reasons are that: (i) the region 300 bps is representative, as the core elements of transcription in eukaryotes are located between −250 bps and +50 bps of TSS [54]; and (ii) both datasets are collated newly by Zhang et al. [48], making the model more reliable and convincing in terms of sample size and data update. For the subsequent model training, all kinds of datasets were split into training sets (including training and validation datasets) and independent test sets with a ratio of 4:1, respectively.

### 2.3. Sequence Feature-Encoding Method

As the basic building blocks of DNA, there are generally four types of nucleotides in eukaryotic organisms. These are Adenine (A), Thymine (T), Guanine (G), and Cytosine (C). Instead of one-hot encoding, we utilized four encoding methods that cover the biochemical and frequency properties of nucleotides in this study. 

#### 2.3.1. Nucleotide Chemical Property (NCP) Feature Encoding

The variety of chemical structures in nucleotides is essential, with ring structures, hydrogen bonds, and functional groups being the most important [55]. Pyrimidines and purines share the six-membered ring as a chemical property. Purines A and G have a pentagon and a hexagon in general, whereas pyrimidines C and T have only one hexagon. In terms of hydrogen bonds, it is worth noting that the number of hydrogen bond donors is unequal, resulting in the formation of three hydrogen bonds between C and G and two between A and T, in turn leading to changes in the strength of hydrogen bonds. Furthermore, ketone groups are closely related to G and T, but A and C are more important for the structural composition of amino.

As in the above manner, one nucleotide n can be encoded by a three-dimensional vector (r, h, f):

(1) $$r=\left\{\begin{array}{c} 0\;\;\;\;if\;n\in \;\left\{C,T\right\}\\ 1\;\;\;if\;n\in \;\left\{A,G\right\} \end{array}\right.,\;\;\;\;h=\left\{\begin{array}{c} 0\;\;\;\;if\;n\in \;\left\{C,G\right\}\\ 1\;\;\;if\;n\in \;\left\{A,T\right\} \end{array}\right.,\;\;\;\;f=\left\{\begin{array}{c} 0\;\;\;\;if\;n\in \;\left\{G,T\right\}\\ 1\;\;\;if\;n\in \;\left\{A,C\right\} \end{array}\right.$$

where r, h, and f represent the NCP encoding result of nucleotide n, respectively.

#### 2.3.2. Nucleotide Density (ND) Feature Encoding

The nucleotide density (ND) can measure the correlation of position and frequency by extracting nucleotide weight information within a sequence [56], which is formally stated as:

(2) $$ND_{n_{i}}=\frac{1}{i}\sum_{k=1}^{i}g\left(n_{k}\right),\;\;\;\;g\left(n_{k}\right)=\left\{\begin{array}{c} 1\;\;\;\;\;\;if\;\;n_{k}=n_{i}\\ 0\;\;\;\;\;\;otherwise \end{array}\right.\;,$$

Here, n_i_ is the i-th nucleotide in a DNA sequence from the first position, and each DNA sequence was represented as a one-dimensional vector.

The NCP- and ND-encoding methods have been successfully applied in bioinformatics such as motif discovery. The existing studies have combined them to form NCP-ND feature encoding, which we will also use in the paper. In NCP-ND feature-encoding methods, a nucleotide within the promoter sequence can be represented as:

(3) $$\left(NCP-ND\right)=\left(r,h,f,nd\right)$$

where r, h, and f indicate the NCP-encoding result of nucleotide n, respectively, and nd is the ND-encoding result of nucleotide n, taking into account both chemical property and element frequency. 

#### 2.3.3. Electron–Ion Interaction Pseudopotential (EIIP) Feature Encoding

By measuring the energy of delocalized electrons in nucleotides, the electron–ion interaction pseudopotential (EIIP) [57] is a single indicator sequence that shows how the free electron energies are distributed throughout the DNA sequence. EIIP represents the nucleotides T, A, G, and C as 0.1335, 0.1260, 0.0806, and 0.1340, respectively, which can reduce the computing cost.

#### 2.3.4. Bi-Profile Bayes (BPB) Feature Encoding

Originally employed for protein methylation sites, the bi-profile Bayes (BPB) [58] encoding system has been widely applied to granzyme cleavage site prediction and enhancer detection. The nucleotide ni for a promoter sequence at the i-th position can be encoded by the BPB-encoding methods as:

(4) $$BPB_{n_{i}}=\left(p^{+},\;\;p^{-}\right)$$

where p^+^ denotes the posterior probability of the nucleotide n_i_ in the positive samples of training datasets, and p^−^ denotes the posterior probability of the nucleotide n_i_ in the negative samples of training datasets.

### 2.4. Graph Construction—Fast Linear Neighborhood Similarity Approach (FLNSA)

The relationships among the entire set of promoter sequences can be described by a graph defined as:

(5) $$G=\left(X,\;E\right),$$

where X = {x_1_, x_2_, …, x_m_} represents the set of nodes, and E = {(i, j)| x_i_ is adjacent to x_j_} is the set of edges. The relation among samples in G can be measured utilizing the fast linear neighborhood similarity approach (FLNSA) [59]. By fusing the NCP-ND, EIIP, and BPB features, a node with the length l can be created. Additionally, there will be an edge in the task if two sample nodes have potential relation.

The dataset’s sequence samples are all converted into vectors x_1_, x_2_, …, x_m_, where x_i_ (1 ≤ i ≤ m) is the i-th sample vector and m is the sample count. The vectors are gathered into an m × l matrix X under the FLNSA assumption that each sample node can be seen as the reconstruction by the linear weighting of other nodes. The following function is optimized by FLNSA for the node construction errors:

$$\begin{array}{c} min\\ w \end{array}\frac{1}{2}\|X-\left(C\;*\;W\right)X\|\begin{array}{c} 2\\ F \end{array}+\frac{u}{2}\sum_{i=1}^{m}\|\left(C\;*\;W\right)e\|\begin{array}{c} 2\\ F \end{array}$$

(6) $$s.t.\;\left(C\;*\;W\right)e=e,\;W\geq 0,$$

where $\|\;{\|}_{F}$ is the Frobenius norm, ∗ represents the Hadamard product, u is the regularization parameter, and e = (1, 1, …, 1)^T^ is an m-dimensional vector with all column values equal to 1. C is an m × m indicator matrix with the element c(i, j) denoted as the following equation:

(7) $$c\left(i,j\right)=\left\{\begin{array}{c} 1,\;\;\;\;\;\;\;\;\;\;\;\;if\;x_{j}\in N\left(X_{i}\right)and\;i\;\neq j\\ 0,\;\;\;\;\;\;\;\;\;\;\;\;\;\;\;\;\;\;\;\;\;\;\;\;\;\;\;\;\;otherwise \end{array}\right.,$$

where $N\left(X_{i}\right)$ is the nearest neighborhood nodes set of $X_{i}$ By determining the neighborhood ratio and calculating the Euclidian distance between $X_{i}$ and other sample node, $N\left(X_{i}\right)$ can be formed. W is an m × m matrix, measuring other nodes’ reconstruction weight contributions to $X_{i}$ in the i-th row. With the Lagrange multiplier method and Karush–Kuhn–Tucker conditions, it can be derivatized that:

(8) $$W_{ij}=\left\{\begin{array}{cc} \frac{W_{ij}{\left(XX^{T}+\mu ee^{T}\right)}_{ij}}{{\left(\left(C\;*\;W\right)XX^{T}+\mu \;\left(C\;*\;W\right)ee^{T}\right)}_{ij}},\;\;\; & x_{j}\in N\;\left(x_{i}\right)\\ 0, & x_{j}\notin N\;\left(x_{i}\right) \end{array}\right.$$

where the matrix W is randomly initialized and iteratively updated until convergence. The adjacency matrix W will construct a graph. The graph-embedding methods require a connected graph as input. For every sample node, FLNSA can find the nearest c (0 < c < m) samples to make the Graph G connected.

### 2.5. Graph-Embedding Feature-Encoding Method

The graph embedding maps each node to a dense, low-dimensional feature vector and tries to preserve the connection strengths and attribute correlations between vertices [60]. We implement three proposed graph-embedding approaches to extract the information from single node, group community, and global structure aspect.

#### 2.5.1. Single Node—Node2vec

Node2vec [61] is a micro-level graph-embedding algorithm that prioritizes the individual. With its focus on a single node, Node2vec applies an upgraded version of the random wandering approach to gather information on how node interacts with its neighbors. 

#### 2.5.2. Group Community—SocDim

A meso-level structural-representation-learning method called SocDim [62] focuses on group interactions. It splits the graph into separate, non-overlapping communities using a modularity strategy. Due to the fact that nodes within a community have those attributes that are different from nodes outside of it, this capture greatly simplifies node analysis and computational learning in graphs.

#### 2.5.3. Global Structure—GraRep

The node vectors produced by GraRep [63] have a global character because of various informational steps reflected in the mapping subspaces. It can generate a low-dimensional vector representation at the scale of the entire graph by taking into account the similarity in long-distance nodes. 

### 2.6. Ensemble-Learning Strategy

Ensemble learning describes a set of strategies that mix numerous “base” models to carry out supervised and unsupervised task, rather than creating a single model. The CatBoost model [64] is a traditional supervised learning ensemble approach. In boosting process, training set is used to generate a classifier with above-average accuracy. And new based classifiers are then added to produce an ensemble, in which the joint decision rules have a higher accuracy level. As a result, the classification performance is improved. 

## 3. Results

### 3.1. Performance Evaluation

To fully verify the effectiveness of our proposed method, six evaluation metrics are used to assess the method’s performance, including sensitivity (Sen), specificity (Spe), accuracy (Acc), Matthew’s correlation coefficient (MCC), precision (Pre), and F1 score. The above six metrics are defined as follows:

(9) $$Sen=\frac{TP}{TP+FN}$$

(10) $$Spe=\frac{TN}{TN+FP}$$

(11) $$Acc=\frac{TP+TN}{TP+FP+TN+FN}$$

(12) $$MCC=\frac{TP\times TN-FP\times FN}{\sqrt{\left(TP+FN\right)\times \left(TN+FP\right)\times \left(TP+FP\right)\times \left(TN+FN\right)}}$$

(13) $$Pre=\frac{TP}{TP+FP}$$

(14) $$F1=\frac{2TP}{2TP+FP+FN}$$

where TP and TN represent the number of true-positive samples and true-negative samples, respectively; and FP and FN represent the number of false-positive samples and false-negative samples, respectively. By the receiver operating characteristic curve (ROC), the area under the curve (AUC) has also been used to measure the performance of the model. In general, the closer the value of AUC is to 1, the more realistic and reliable the overall performance is.

### 3.2. Performance on Balanced Datasets

In this section, we tested models on balanced datasets, as shown in Table 3.

For *H. sapiens* (251 bps), PromGER was compared with CNNProm [37], NNPP2.2 [20], and FProm [45] on the independent test dataset. As a result, PromGER acquired the best metrics for both TATA-containing and TATA-less promoters with the exception of Spe and Pre, which were only 0.0043 and 0.0028 lower than that of CNNProm. Compared with the suboptimal predictor CNNProm, PromGER improved the AUC of 0.0702 for predicting TATA-containing promoters, and 0.086 for TATA-less promoters, respectively. 

For *H. sapiens* (300 bps), PromGER was compared with iProEP [27], Depicter [39], and DeePromoter [38] on the independent test dataset. We can observe that PromGER had the best performance in terms of all major measurements among these predictors. The Sen of PromGER is 0.0494 and 0.0244 lower than the suboptimal value of the predictor Depicter for TATA-containing promoters and DeePromoter for TATA-less promoters, respectively. 

For *R. norvegicus* (251 bps), PromGER was compared with CNNProm and NNPP2.2 on the independent test dataset because FProm did not consider this situation. PromGER achieved the highest Acc, MCC, F1, and AUC on both TATA-containing and TATA-less datasets. Its AUC is 0.9923 and 0.9488, which outperformed other methods.

For *R. norvegicus* (300 bps), PromGER was compared with Depicter and DeePromoter on the independent test dataset, because iProEP did not consider this situation. PromGER is also superior to other baselines by achieving an Spe of 0.945, Acc of 0.9395, MCC of 0.8791, Pre of 0.9444, F1 of 0.9392, and AUC of 0.9841 for TATA-containing sequences. Moreover, PromGER has improved slightly in all six measures except Sen on the TATA-less dataset.

### 3.3. Performance on Imbalanced Datasets

In this section, we tested models on imbalanced datasets, as shown in Table 4.

On the *H. sapiens* (251 bps) dataset, PromGER obtained the highest metrics except Sen for the TATA-containing promoters, which was 0.0854 lower than NNPP2.2. For the TATA-less promoters, PromGER performed the overall best with AUC (0.9932). As the crucial evaluation for imbalanced datasets, PromGER improved its predictive performance with MCC and F1. 

On the *H. sapiens* (300 bps) dataset, PromGER also performed well. Compared with other predictors, PromGER predicted better with an Acc of 0.9338 and 0.9560 on the TATA-containing and TATA-less datasets, respectively. Its MCC and F1 were slightly lower than Depicter.

On the *R. norvegicus* (251 bps) and *R. norvegicus* (300 bps) datasets, PromGER achieved a better performance than Depicter and DeePromoter in terms of Spe, Acc, MCC, Pre, F1, and AUC. The Sen of PromGER was 0.0228 lower and 0.0286 lower than CNNProm and Depicter on the TATA-containing datasets of 251 bps and 300 bps, respectively. For TATA-less datasets of 300 bps, PromGER was 0.0353 lower than Depicter.

**Figure 2:**
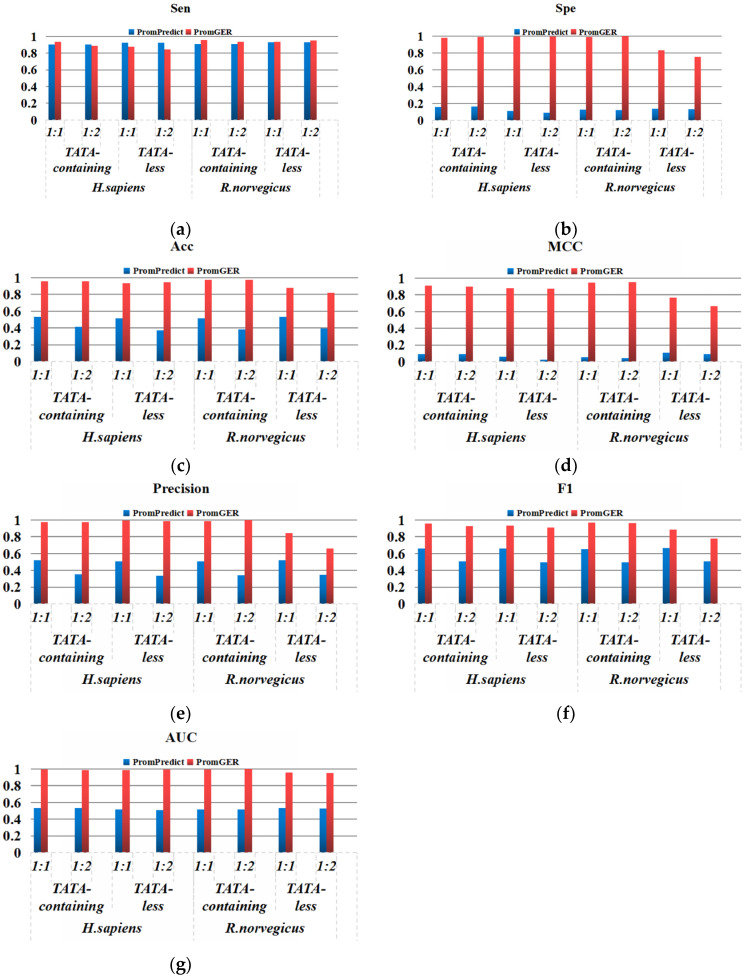
Performance comparison between PromGER and PromPredict (1001 bps) on the independent test sets, including balanced and imbalanced: (**a**) Sen; (**b**) Spe; (**c**) Acc; (**d**) MCC; (**e**) Pre; (**f**) F1; and (**g**) AUC.

We also took into account a model comparison of 1001 bps [46] in addition to the 251 bps and 300 bps input, though such tools were uncommon. As shown in Figure 2, PromGER still maintained greater stability for longer sequences.

### 3.4. Ablation Study of Different Feature Combinations

**Figure 3:**
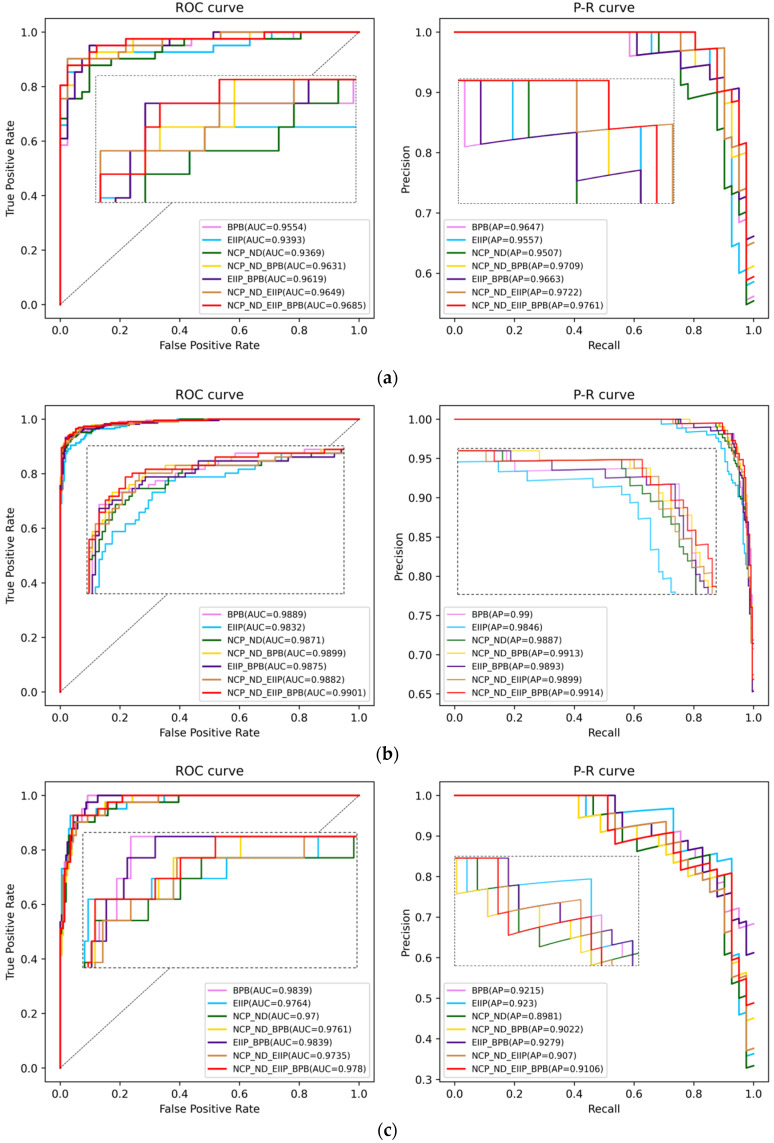

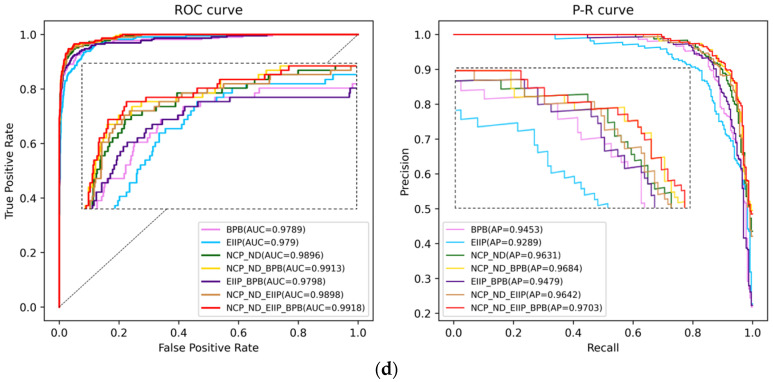
The ROC and PR curves of the comparison results: (**a**) the curves on the balanced TATA-containing dataset; (**b**) the curves on the balanced TATA-less dataset; (**c**) the curves on the imbalanced TATA-containing dataset; and (**d**) the curves on the imbalanced TATA-less dataset.

In order to verify the effectiveness of the sequence features on promoter prediction, we focused on seven different feature combinations as the input of the PromGER. All features are removed before the graph embedding, including NCP_ND, EIIP, and BPB. On the independent test datasets of *D. melanogaster*, we used more extreme imbalanced data besides balanced datasets, where the ratio of positive samples to negative samples (i.e., promoters: non-promoters) is 1:5. We introduced the Kappa coefficient as a measure to capture the consistency:

(15) $$K_{appa}=\frac{Acc-\frac{a_{1}\times b_{1}+a_{2}\times b_{2}+\cdots +a_{m}\times b_{m}}{n\times n}}{1-\frac{a_{1}\times b_{1}+a_{2}\times b_{2}+\cdots +a_{m}\times b_{m}}{n\times n}}$$

where n is the total number of samples; a_m_ denotes the actual sample number in class m, and b_m_ denotes the predicted sample number in class m, accordingly. Table 5 shows the comparison results, with the receiver operating characteristic curve (ROC) and the precision-recall (PR) curve displayed in Figure 3.

For the balanced datasets, the Kappa coefficient of NCP_ND + EIIP + BPB was increased by 16.65% and 0.23% compared with the worst (only NCP_ND) and the suboptimality (EIIP + BPB) on the TATA-containing type. Meanwhile, on the TATA-less type, although the trend was similar to the TATA-containing type, the improvement was caused by EIIP encoding and NCP_ND + BPB, respectively.

For the imbalanced dataset, the Kappa coefficient of NCP_ND + EIIP + BPB was increased by 7.15% and 1.88% compared with the worst (NCP_ND + EIIP) and the suboptimality (only NCP_ND) on the TATA-containing type, but the ROC curve and the PR curve showed minor fluctuations. On the TATA-less type, NCP_ND + EIIP + BPB was better in all aspects.

Regarding the combination of graph-embedding features, we also design the ablation experiments shown in Table 6. For the balanced datasets, the Kappa coefficient of Node2vec + SocDim + GraRep was increased by 8.48% compared with the worst (only SocDim) and 0.45% compared with the suboptimality (Node2vec + SocDim). Meanwhile, on the TATA-less type, the corresponding results are 4.81% (only Node2vec) and 1.56% (only GraRep). For the imbalanced datasets, the Kappa coefficient of Node2vec + SocDim + GraRep was increased by 2.99% compared with the worst (only SocDim) and 0.05% compared with the suboptimality (only GraRep). Likewise, on the TATA-less type, the corresponding results are 3.41% (only GraRep) and 0.49% (Node2vec + SocDim). Therefore, it is helpful for the prediction to extract the graph-embedding information from the single node, group community, and global structure aspect.

### 3.5. Model Visualization Interpretation

Deep-learning and machine-learning methods are the “black box”, which is not visible for the hidden intermediate process. In other words, it is beneficial for the model reality to improve the interpretability of such models, which has been the major target of research efforts. To provide an explanation for the predictive behavior of PromGER, we employed a visualization algorithm called t-SNE. The t-SNE maps data from the high-dimensional feature space to the two-dimensional space by nonlinear dimensionality reduction. To further understand the prediction results of PromGER, we introduced the SHAP value analysis to enhance an interpretation of the decision results.

#### 3.5.1. Visualization in Graph-Embedding Period

**Figure 4:**
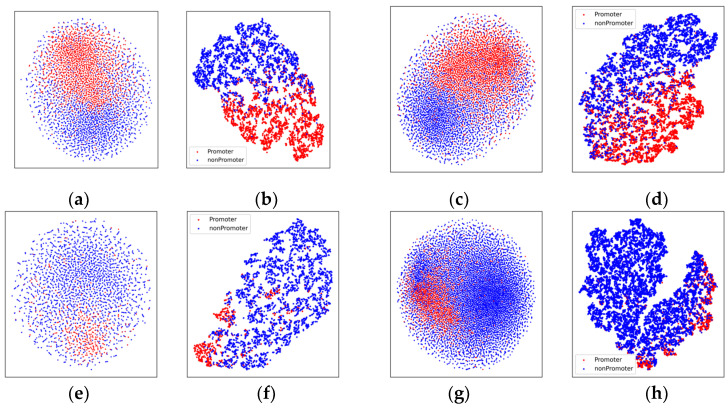
The t-SNE visualization results on the *Zea mays* datasets, in which red dots are promoters and blue dots are non-promoters, respectively (perplexity: 15): (**a**,**b**) the results on the balanced TATA-containing dataset; (**c**,**d**) the results on the balanced TATA-less dataset; (**e**,**f**) the results on the imbalanced TATA-containing dataset; and (**g**,**h**) the results on the imbalanced TATA-less dataset. (**a**,**c**,**e**,**g**) represent the results before graph embedding. (**b**,**d**,**f**,**h**) represent the results after graph embedding.

The comparison results on the *Z. mays* datasets are shown in Figure 4. Before graph embedding, the two types of points are mixed together. However, the feature vectors can be well-separated after graph-embedding processing.

#### 3.5.2. Visualization in Ensemble-Learning Periods

**Figure 5:**
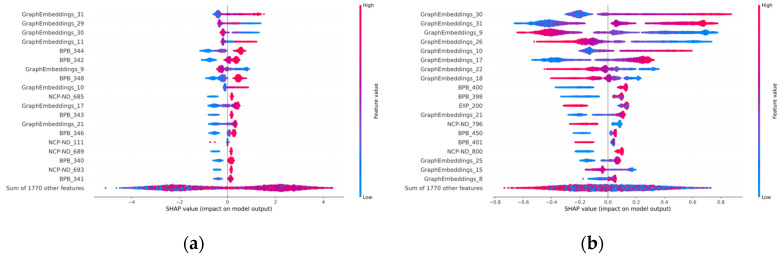

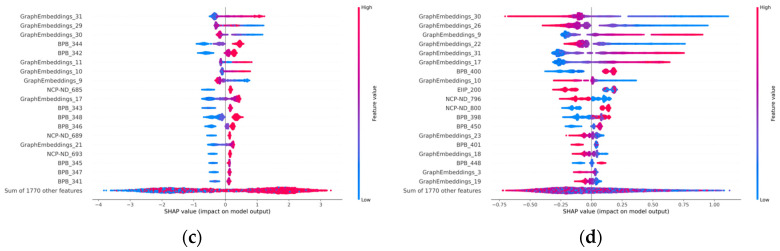
SHAP visualization results on the *Zea mays* datasets, in which positive SHAP values indicate positive sample predictions, and negative SHAP values indicate negative sample predictions (blue colors: low feature values; and red colors: high feature values): (**a**) the results on the balanced TATA-containing dataset; (**b**) the results on the balanced TATA-less dataset; (**c**) the results on the imbalanced TATA-containing dataset; and (**d**) the results on the imbalanced TATA-less dataset.

The SHAP value was a universal measure of feature importance from the coalitional game theory, which provided an importance value for each feature in the ensemble learning. It made the predictions comprehensible by assessing the effect of each feature on the model training. To identify which features are more crucial for PromGER, we used the SHAP value on the *Z. mays* datasets. Figure 5 showed the results based on SHAP values for both TATA-containing and TATA-less types of promoters, revealing the top features ranked by the sum of SHAP-value magnitudes over all samples for both balanced and imbalanced types, which emphasizes the distribution of each feature’s influence on the PromGER output. The results showed that, on the four datasets, PromGER is based on slightly different important features, including both the sequence’s local features and graph-embedded features. This indicates that these two different types of features jointly influence the prediction results. Meanwhile, GraphEmbeddings_31 shows a high positive correlation on different datasets, and BPB_400 shows a negative correlation on TATA-less datasets. Moreover, the top features for the TATA-less prediction are several GraphEmbedding features, implying the importance of graph-embedding methods for the TATA-less promoter prediction.

## 4. Discussion

Promoters are crucial components of the DNA’s non-coding regions, which contribute to exploration in the context of human diseases. Thus, predicting eukaryotic promoter sequences in bioinformatics is valuable for the comprehension of the transcriptional control in genes. Various methods based on machine learning and deep learning have been applied to the promoter prediction tasks in recent years. In this study, we propose a promoter prediction model to predict eukaryotic promoter sequences, which is called PromGER. PromGER obtained the four attribute features of nucleotides within each promoter sequence, including physical features, biochemical properties, molecular characteristics, and frequency distributions. In addition, we also gained a sequence-to-sequence relationship representation by graph-embedding methods. This multi-scale feature extraction approach is able to process deeper semantic information and biological meaning in promoter sequences, compared with most of other models.

We evaluated PromGER on different datasets, and the results demonstrated PromGER obtained the best performance compared to the comparison models. Different from models based on CNN, PromGER has no input sequence constraints, which makes it more generalizable to promoter prediction tasks, regardless of input length or species variations. Our models outperformed the existing models for Acc, MCC, Pre, F1, and AUC, suggesting that the PromGER is a robust and reliable predictor. Meanwhile, the Sen of the PromGER sometimes has a gap with the optimal models. For these models, they tend to extract the intuitive and shallow features like core promoter elements at absolute advantages. However, the fundamental features at specific positions, such as the TATA box, are not a determinant. Furthermore, it may make the model depend on the presence or absence of these local elements to discriminate the promoter, reducing the generalization of the model with a low Spe value. Accordingly, PromGER can achieve a balance between Sen and Spe. More importantly, PromGER extracts the potential relationship among promoter sequences ignored by existing models. For a promoter sequence, three different scales of graph-embedding methods are applied to represent the global features in a graph. We can see that, in addition to the TATA-containing type datasets, PromGER also achieves better results on the TATA-less type datasets. This is perhaps just because potential connections between sequences will contribute to correct predictions using graph-embedding methods. Attributed to CatBoost ensemble learning, PromGER maintained a superior performance on the imbalanced dataset. Through the ablation study for different features, we verified the validity of the encoding. The results indicated that a single feature is not beneficial and feature combination is a very important factor for the prediction. Finally, we provided the visual interpretation of the model with the t-SNE algorithm and SHAP values, which demonstrated these features are effective for predicting different types of promoters and improved the prediction performance of PromGER. And the information within the local features can be complemented by the features from the graph embedding, implying the powerful potential of graph deep learning.

## 5. Conclusions

In this article, we proposed a novel computational model called PromGER to predict the eukaryotic promoters, by combining graph embedding and ensemble learning. The experimental results show that PromGER can predict the eukaryotic promoters accurately for both TATA-containing and TATA-less types. This approach may play a crucial role in supplementing other existing methods of predicting promoters and other biological sites. Future research will consider the potential impact of global information more carefully, and we can expect further improvements by introducing more graph deep-learning methods.

## Figures and Tables

**Table 1:** The balanced datasets for four species.

Species	TATA	Number of Promoters	Number of Non-Promoters	Location	Length
*Homo sapiens*	TATA-containing	229	229	[−200, +50]	251
				[−249, +50]	300
				[−500, +500]	1001
	TATA-less	1328	1328	[−200, +50]	251
				[−249, +50]	300
				[−500, +500]	1001
*Rattus norvegicus*	TATA-containing	456	456	[−200, +50]	251
				[−249, +50]	300
				[−500, +500]	1001
	TATA-less	3834	3834	[−200, +50]	251
				[−249, +50]	300
				[−500, +500]	1001
*Drosophila melanogaster*	TATA-containing	207	207	[−249, +50]	300
	TATA-less	1153	1153	[−249, +50]	300
*Zea mays*	TATA-containing	3051	3051	[−249, +50]	300
	TATA-less	5210	5210	[−249, +50]	300

**Table 2:** The imbalanced datasets for four species.

Species	TATA	Number of Promoters	Number of Non-Promoters	Location	Length
*Homo sapiens*	TATA-containing	229	458	[−200, +50]	251
				[−249, +50]	300
				[−500, +500]	1001
	TATA-less	1328	2656	[−200, +50]	251
				[−249, +50]	300
				[−500, +500]	1001
*Rattus norvegicus*	TATA-containing	456	912	[−200, +50]	251
				[−249, +50]	300
				[−500, +500]	1001
	TATA-less	3834	7668	[−200, +50]	251
				[−249, +50]	300
				[−500, +500]	1001
*Drosophila melanogaster*	TATA-containing	207	1035	[−249, +50]	300
	TATA-less	1153	5765	[−249, +50]	300
*Zea mays*	TATA-containing	610	3050	[−249, +50]	300
	TATA-less	2762	13,810	[−249, +50]	300

**Table 3:** The prediction performance on 251 bps and 300 bps balanced testing datasets.

Datasets		Models	Sen	Spe	Acc	MCC	Pre	F1	AUC
*H. sapiens* (251 bps)	TATA- containing	CNNProm	0.8559	0.9825	0.9192	0.8452	0.9800	0.9138	0.9250
		NNPP2.2	0.9520	0.4279	0.6900	0.4461	0.6246	0.7543	0.6900
		FProm	0.4978	0.9738	0.7358	0.5363	0.9500	0.6533	0.7358
		PromGER	0.9565	0.9782	0.9673	0.9350	0.9772	0.9670	0.9952
	TATA- less	CNNProm	0.8504	0.7995	0.8164	0.6212	0.6791	0.7551	0.8984
		NNPP2.2	0.7417	0.3742	0.5580	0.1247	0.5424	0.6266	0.5580
		FProm	0.4834	0.9571	0.7203	0.5002	0.9185	0.6334	0.7203
		PromGER	0.8646	0.9849	0.9248	0.8558	0.9829	0.9199	0.9844
*H. sapiens* (300 bps)	TATA- containing	iProEP	0.7249	0.9476	0.8362	0.6898	0.9326	0.8157	0.9416
		Depicter	0.9607	0.8996	0.9301	0.8619	0.9053	0.9322	0.9715
		DeePromoter	0.9520	0.4279	0.6900	0.4461	0.6246	0.7543	0.6900
		PromGER	0.9113	0.9757	0.9424	0.8908	0.9761	0.9425	0.9975
	TATA- less	iProEP	0.6190	0.9262	0.7726	0.5729	0.8935	0.7313	0.8589
		Depicter	0.7869	0.8253	0.8061	0.6127	0.8183	0.8023	0.8446
		DeePromoter	0.9149	0.1453	0.5301	0.0943	0.5170	0.6607	0.5301
		PromGER	0.8905	0.9849	0.9377	0.8793	0.9833	0.9346	0.9832
*R. norvegicus* (251 bps)	TATA- containing	CNNProm	0.9298	0.9649	0.9474	0.8953	0.9636	0.9464	0.9596
		NNPP2.2	0.9452	0.4232	0.6842	0.4319	0.6210	0.7496	0.6842
		PromGER	0.9350	0.9670	0.9560	0.9123	0.9662	0.9555	0.9923
	TATA- less	CNNProm	0.8263	0.8905	0.8584	0.7182	0.8829	0.8537	0.8883
		NNPP2.2	0.7433	0.4520	0.5977	0.2042	0.5756	0.6488	0.5977
		PromGER	0.9425	0.7819	0.8596	0.7281	0.8112	0.8697	0.9488
*R. norvegicus* (300 bps)	TATA- containing	Depicter	0.9846	0.7522	0.8684	0.7576	0.7989	0.8821	0.9786
		DeePromoter	0.8158	0.4781	0.6469	0.3122	0.6098	0.6979	0.6469
		PromGER	0.9340	0.9450	0.9395	0.8791	0.9444	0.9392	0.9841
	TATA- less	Depicter	0.9726	0.7467	0.8597	0.7384	0.7934	0.8739	0.8839
		DeePromoter	0.8192	0.2433	0.5313	0.0766	0.5199	0.6361	0.5313
		PromGER	0.9516	0.7806	0.8661	0.7433	0.8127	0.8767	0.9485

**Table 4:** The prediction performance on 251 bps and 300 bps imbalanced testing datasets (promoters:non-promoters = 1:2).

Datasets		Models	Sen	Spe	Acc	MCC	Pre	F1	AUC
*H. sapiens* (251 bps)	TATA- containing	CNNProm	0.8518	0.8686	0.8602	0.7205	0.8664	0.8590	0.8979
		NNPP2.2	0.9520	0.4279	0.6026	0.3903	0.4542	0.6150	0.6900
		FProm	0.4978	0.8996	0.7656	0.4432	0.7125	0.5861	0.6987
		PromGER	0.8666	0.9780	0.9411	0.8661	0.9512	0.9069	0.9736
	TATA- less	CNNProm	0.8124	0.5549	0.7089	0.4391	0.6597	0.7477	0.7089
		NNPP2.2	0.7417	0.3852	0.5040	0.1260	0.3762	0.4992	0.5634
		FProm	0.4834	0.8991	0.7605	0.4295	0.7055	0.5737	0.6913
		PromGER	0.8679	0.9962	0.9535	0.8961	0.9913	0.9255	0.9932
*H. sapiens* (300 bps)	TATA- containing	iProEP	0.7249	0.9585	0.8806	0.7263	0.8973	0.8019	0.9495
		Depicter	0.9607	0.9170	0.9316	0.8544	0.8527	0.9035	0.9438
		DeePromoter	0.9520	0.4760	0.6346	0.4279	0.4760	0.6346	0.7140
		PromGER	0.8666	0.9670	0.9338	0.8490	0.9285	0.8965	0.9755
	TATA- less	iProEP	0.6190	0.9326	0.8281	0.5995	0.8212	0.7059	0.8576
		Depicter	0.7869	0.7952	0.7924	0.5604	0.6576	0.7165	0.7640
		DeePromoter	0.9149	0.1224	0.3865	0.0562	0.3426	0.4986	0.5186
		PromGER	0.8754	0.9962	0.9560	0.9017	0.9914	0.9298	0.9871
*R. norvegicus* (251 bps)	TATA- containing	CNNProm	0.9578	0.9641	0.9610	0.9220	0.9639	0.9608	0.9757
		NNPP2.2	0.9452	0.4243	0.5980	0.3797	0.4508	0.6105	0.6848
		PromGER	0.9350	0.9945	0.9780	0.9505	0.9885	0.9662	0.9972
	TATA- less	CNNProm	0.6934	0.5415	0.6174	0.2376	0.5519	0.6444	0.6174
		NNPP2.2	0.7433	0.4384	0.5401	0.1768	0.3983	0.5187	0.5909
		PromGER	0.9451	0.6784	0.7672	0.5888	0.5949	0.7302	0.9290
*R. norvegicus* (300 bps)	TATA- containing	Depicter	0.9846	0.8169	0.8728	0.7595	0.7289	0.8377	0.9267
		DeePromoter	0.8158	0.4386	0.5643	0.2508	0.4208	0.5552	0.6272
		PromGER	0.9560	0.9835	0.9743	0.9421	0.9666	0.9613	0.9952
	TATA- less	Depicter	0.9726	0.7851	0.8476	0.7159	0.6935	0.8097	0.8480
		DeePromoter	0.8192	0.2237	0.4222	0.0497	0.3454	0.4859	0.5215
		PromGER	0.9373	0.9680	0.9578	0.9050	0.9361	0.9367	0.9902

**Table 5:** The Kappa coefficient comparison results by using different sequence feature combinations on *D. melanogaster* datasets.

Feature Combinations			Kappa			
			Promoters:Non-Promoters = 1:1		Promoters:Non-Promoters = 1:5	
NCP_ND	EIIP	BPB	TATA-Containing	TATA-Less	TATA-Containing	TATA-Less
√			0.7317	0.8913	0.8189	0.8699
	√		0.8048	0.8608	0.8043	0.8027
		√	0.8495	0.8826	0.8031	0.8207
√	√		0.7804	0.8869	0.7786	0.8801
	√	√	0.8516	0.8913	0.7993	0.8421
√		√	0.7804	0.8956	0.8031	0.8572
√	√	√	0.8536	0.9130	0.8343	0.8833

**Table 6:** The Kappa coefficient comparison results by using different graph-embedding feature combinations on *D. melanogaster* datasets.

Feature Combinations			Kappa			
			Promoters:Non-Promoters = 1:1		Promoters:Non-Promoters = 1:5	
Node2vec	SocDim	GraRep	TATA-Containing	TATA-Less	TATA-Containing	TATA-Less
√			0.8591	0.8958	0.8415	0.8908
	√		0.8390	0.9079	0.8213	0.8838
		√	0.8835	0.9283	0.8507	0.8627
√	√		0.9193	0.9097	0.8498	0.8919
	√	√	0.8611	0.9205	0.8451	0.8776
√		√	0.8730	0.9113	0.8364	0.8878
√	√	√	0.9238	0.9439	0.8512	0.8968

## Data Availability

Publicly available datasets include detailed information about PromGER, which can be downloaded at: https://github.com/XiaoxiangHugh/PromGER accessed on 8 August 2022.
